# Antitumor and anti-angiogenic potentials of isolated crude saponins and various fractions of *Rumex hastatus* D. Don.

**DOI:** 10.1186/s40659-016-0079-2

**Published:** 2016-03-12

**Authors:** Sajjad Ahmad, Farhat Ullah, Muhammad Ayaz, Anwar Zeb, Farman Ullah, Abdul Sadiq

**Affiliations:** Department of Pharmacy, University of Malakand, Chakdara, Dir (L), Khyber-Pakhtunkhwa (KPK) 18000 Pakistan; Department of Pharmacy, Kohat University of Science and Technology, Kohat, Khyber-Pakhtunkhwa (KPK) 26000 Pakistan

**Keywords:** *Rumex hastatus*, *Agrobacterium tumefaciens*, Saponins, Angiogenesis, Tumor

## Abstract

**Background:**

Cancer, being the foremost challenge of the modern era and the focus of world-class investigators, gargantuan research is in progress worldwide to explore novel therapeutic for its management. The exploitation of natural sources has been proven to be an excellent approach to treat or minify the excessive angiogenesis and proliferation of cells. Similarly, based the ethnomedicinal uses and literature survey, the current study is designed to explore the anti-tumor and anti-angiogenic potentials of *Rumexhastatus*. Anti-tumor and anti-angiogenic activities were carried out using potato-disc model and chorioallantoic membrane (CAM) assay respectively. Moreover, *R. hastatus* was also assessed for antibacterial activity against *Agrobacteriumtumefaciens* (tumor causing bacterial strain). The positive controls used in anti-tumor, anti-angiogenic and antibacterial activities were vincristine sulphate, dexamethasone and cefotaxime respectively.

**Results:**

The crude saponins (Rh.Sp), methanolic extract (Rh.Cr) and other solvent extracts like *n*-hexane (Rh.Hex), chloroform (Rh.Chf), ethylacetate (Rh.EtAc) and aqueous fraction (Rh.Aq) exhibited notable anti-tumor and anti-angiogenic activities. In potato tumor assay, the chloroform and saponin fractions were observed to be the most effective showing 86.7 and 93.3 % tumor inhibition at 1000 µg/ml with IC_50_ values 31.6 and 18.1 µg/ml respectively. Similarly, these two samples i.e., chloroform and saponins also excelled among the entire test samples in anti-angiogenic evaluation exhibiting 81.6 % (IC_50_ = 17.9 µg/ml) and 78.9 % (IC_50_ = 64.9 µg/ml) at 1000 µg/ml respectively. In contrast, the antibacterial investigations revealed a negligible potential against *A. tumefaciens*.

**Conclusion:**

Based on our results we can claim that *R. hastatus* possesses both anti-tumor and anti-angiogenic potentials. In all of the solvent fractions, Rh.Chf and Rh.Sp were most effective against tumor and angiogenesis while having negligible activity against *A. tumefaciens*. It can be concluded that Rh.Chf and Rh.Sp might be potential targets in the isolation of natural product having anti-neoplastic action.

## Background

In each and every era, the world has to face various challenges. The challenge to survive and to combat various health anomalies is the foremost challenge of each era. Similarly, in the context of health anomalies, tumor is the most challenging threat to the current era [1]. Tumor is mainly characterized by abnormal and excessive proliferation of cells, which progressively overrun and disrupt the neighboring cells. The angiogenesis i.e., formation of new blood vessels also occurs along with the proliferation of cells which rarely occurs in normal tissues except in the wound healing and embryogenesis [2]. It has been obviously manifested that excessive angiogenesis leads to several pathological conditions including cancer, atherosclerosis, arthritis, ovarian cyst and osteomyelitis [3]. Tumor progressions always require increase in number of blood vessels and similarly decrease in number of blood vessels in milieu leads to dormancy of tumor. Angiogenic pathway is a sound target to obstruct the excessive proliferation of cells because the nutrients and growth factors are supplied through blood vessels to the tumor cells. Anti-angiogenesis is probably one of the leading strategies of the emerging oncologists to combat cancer [4]. Several chemotherapeutic agents are used against the angiogenesis-dependent pathophysiological conditions especially against tumor. The synthetic chemotherapeutic agents being associated with plethora of hazardous effects are discouraged and the investigators are trying to explore bioactive agents derived from natural sources to cure tumor and other lethal diseases [5–7]. A leading source of natural bioactive compounds i.e., plants have been gaining much more attention of the researchers for their good efficacy and low toxicity [8, 9].

Potato tumor assay has been conducted on several plants of various families with prominent results [10, 11]. Similarly, strong anti-angiogenic activity has also been demonstrated by several plant species following chorioallantoic membrane (CAM) assay [12, 13]. Numerous bioactive compounds have been isolated from various plants and have been evaluated against tumor with good results [14].

In the plants kingdom several families and genera have been evaluated against tumor and one of the genera i.e., *Rumex* has been proven to be a good source of antitumor compounds [15–17]. Several compounds have been isolated from *Rumex hymenosepalus* having antitumor potential [18]. Moreover, the *Rumex* species are being used in wound healing, astringent [19, 20], anti-asthmatic, antitussive, anti-tumor and antioxidant [1]. It is evident that almost all the species of a specific genus resembles considerably due to genome similarity among the species of the same genus [21]. Similarly, *R.hastatus* has been traditionally used against pimples, wounds, scorpion stings, foot and mouth diseases, eye diseases, giddiness and insanity [22]. It has also been reported to be used traditionally as laxative, alterative, tonic, in rheumatism [23], in skin diseases, piles, bilious complaints and lungs bleeding [24]. The juice of *R. hastatus* is used to treat blood pressure [25], in tonsillitis, sore throat [26], as flavoring agent, carminative and diuretic [27]. Moreover, this plant has recently been reported with excellent pharmacological activities [28, 29].

Based on the literature survey and ethnomedicinal relevance of *R. hastatus*, this study was designed to evaluate the anti-tumor and anti-angiogenic potentials of *R. hastatus* and to corroborate this plant as a possible remedy of neoplasia.

## Results

### Anti-tumor effect

**Fig. 1:**
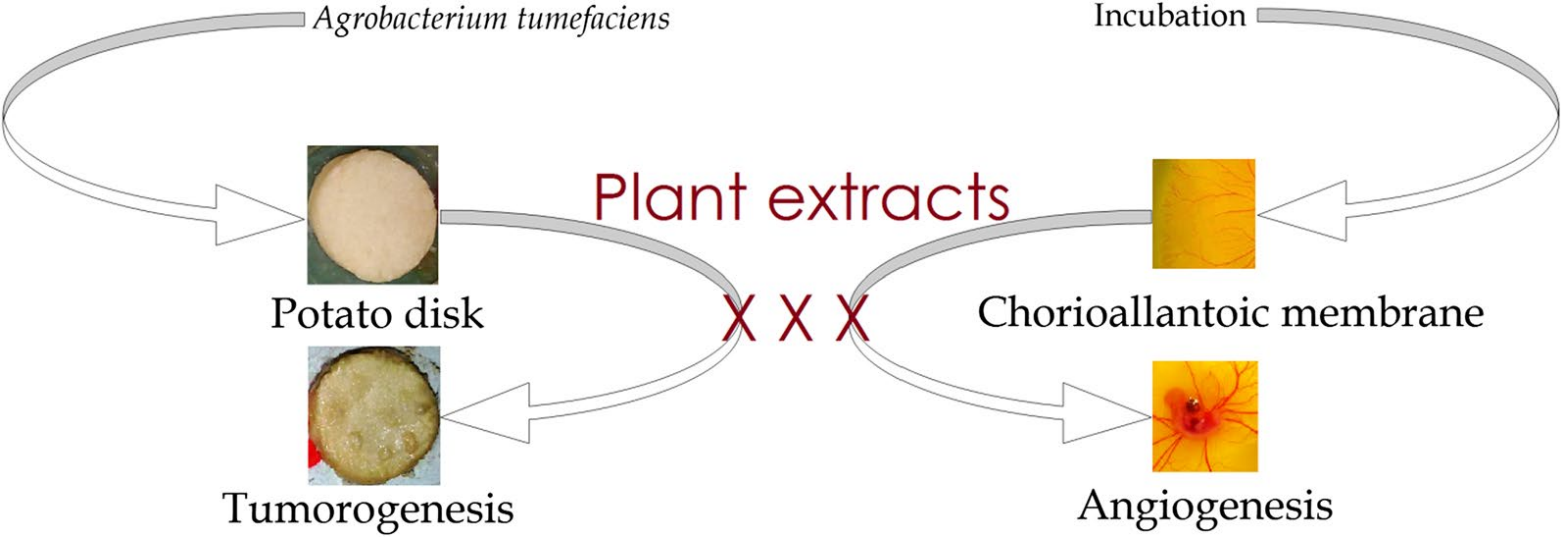
Graphical presentation of potato disc anti-tumor assay (*Left*) and CAM angiogenesis assay (*Right*)

**Fig. 2:**
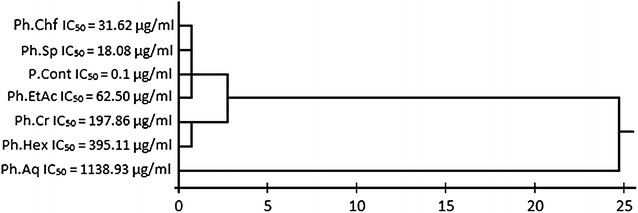
Cluster analysis and dendrogram based IC50 values of various samples of *Rumex* *hastatus* in anti-tumor assay. *Rh.Cr* Crude methanolic extract, *Rh.Hex* *n*-hexane fraction, *Rh.Chf* chloroform fraction, *Rh.EtAc* ethyl acetate fraction, *Rh.Aq* aqueous fraction, *Rh.Sp* saponins, *P.Cont* positive control

**Fig. 3:**
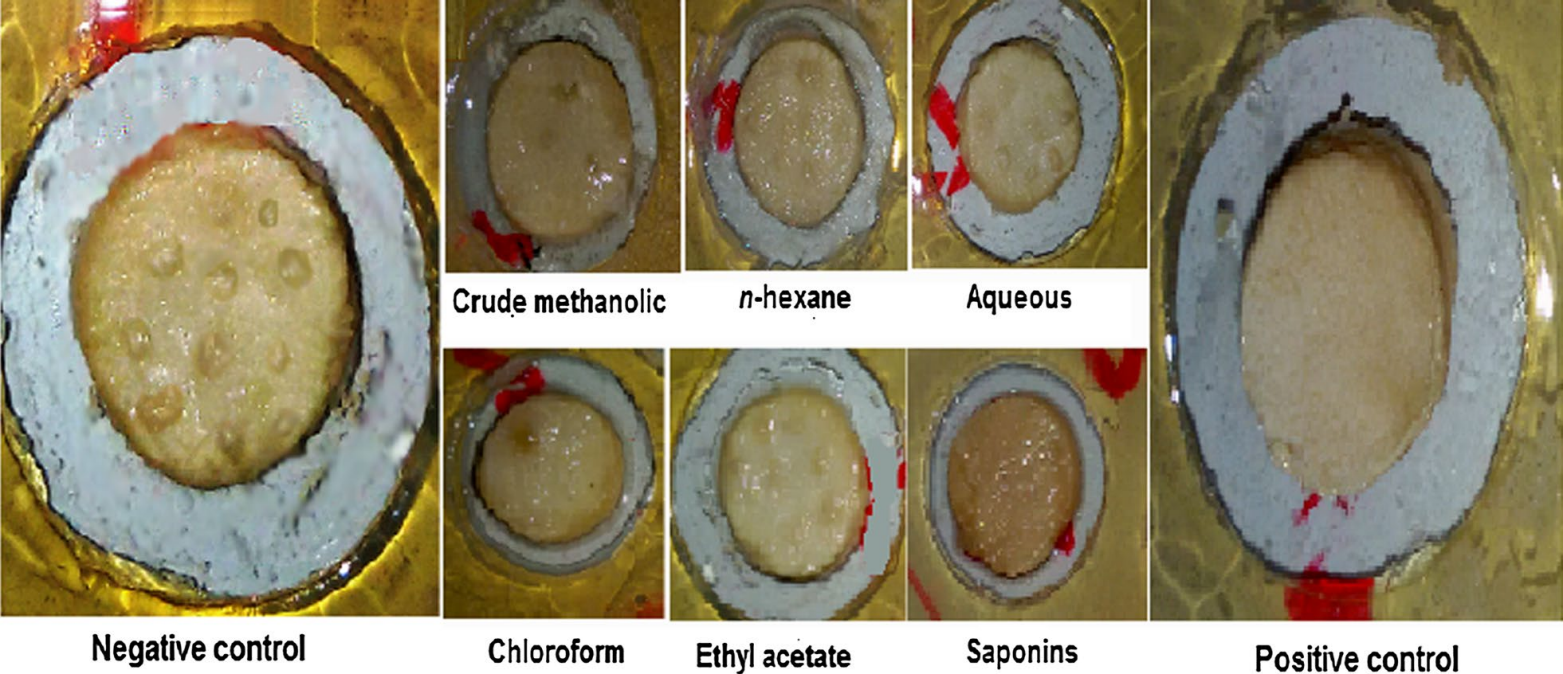
Antitumor activity of various samples of *Rumex hastatus* along with positive and negative controls. Positive control used was applied with vincristine sulphate and *Agrobacterium tumefaciens* while the negative control was only applied with *Agrobacterium tumefaciens*

A general graphical picture based on our potato anti-tumor and CAM angiogenesis results is summarized as Fig. 1. The anti-tumor assay carried out for various samples of *R. hastatus* revealed a dose dependent anti-tumor response (Table 1). Among the test samples, the saponins showed excellent anti-tumor activity i.e., 93.3 ± 0.0, 83.3 ± 1.9, 78.9 ± 1.1, 63.3 ± 1.9, 56.6 ± 3.8 and 53.3 ± 0.0 % at concentrations of 1000, 500, 250, 125, 62.5 and 31.25 µg/ml respectively with IC_50_ value of 18.1 µg/ml. The second highest activity has been attributed to chloroform fraction i.e., 86.7 ± 3.8, 77.8 ± 1.1, 70.0 ± 0.0, 65.5 ± 1.1, 58.9 ± 2.2 and 50.0 ± 1.9 % at 1000, 500, 250, 125, 62.5 and 31.25 µg/ml respectively with IC_50_ of 31.6 µg/ml. Similarly at 1000 µg/ml, the Rh.Cr, Rh.Hex, Rh.Chf, Rh.EtAc and Rh.Aq exhibited 85.5 ± 1.1, 63.3 ± 1.9, 86.7 ± 3.8, 80.0 ± 1.9 and 46.7 ± 0.0 % tumor inhibitions respectively. A brief summary of cluster analysis of IC_50_ values are given in Fig. 2 and pictures from the original results of different tested fractions are represented as Fig. 3.

**Table 1:** Anti-tumor activity of various samples of *Rumex* *hastatus*

Samples	Concentrations (µg/ml)	Average inhibition (mean ± SEM)	Percent inhibition (mean ± SEM)	IC_50_ (µg/ml)
Rh.Cr	1000	25.7 ± 0.3	85.5 ± 1.1***	197.9
	500	19.0 ± 0.6	63.3 ± 1.9***	
	250	16.0 ± 1.2	53.3 ± 3.8***	
	125	13.3 ± 0.3	44.4 ± 1.1***	
	62.5	12.0 ± 0.0	40.0 ± 0.0***	
	31.25	10.0 ± 0.6	33.3 ± 1.9***	
Rh.Hex	1000	19.0 ± 0.6	63.3 ± 1.9***	395.1
	500	16.3 ± 0.3	54.4 ± 1.1***	
	250	12.7 ± 0.3	42.2 ± 1.1***	
	125	11.0 ± 1.2	36.7 ± 3.8***	
	62.5	10.7 ± 0.3	35.5 ± 1.1***	
	31.25	08.0 ± 0.0	26.7 ± 0.0***	
Rh.Chf	1000	26.0 ± 1.2	86.7 ± 3.8*	31.6
	500	23.3 ± 0.3	77.8 ± 1.1**	
	250	21.0 ± 0.0	70.0 ± 0.0***	
	125	19.7 ± 0.3	65.5 ± 1.1**	
	62.5	17.7 ± 0.7	58.9 ± 2.2***	
	31.25	15.0 ± 0.6	50.0 ± 1.9***	
Rh.EtAc	1000	24.0 ± 0.6	80.0 ± 1.9**	62.5
	500	19.7 ± 0.7	65.5 ± 2.2***	
	250	18.0 ± 0.6	60.0 ± 1.9***	
	125	16.0 ± 1.2	53.3 ± 3.8***	
	62.5	15.0 ± 0.0	50.0 ± 0.0***	
	31.25	13.0 ± 1.2	43.3 ± 3.8***	
Rh.Aq	1000	14.0 ± 0.0	46.7 ± 0.0***	1138.9
	500	12.6 ± 0.3	42.2 ± 1.1***	
	250	11.6 ± 0.3	38.9 ± 1.1***	
	125	09.0 ± 0.6	30.0 ± 1.9***	
	62.5	08.7 ± 0.3	28.9 ± 1.1***	
	31.25	05.3 ± 0.3	17.8 ± 1.1***	
Rh.Sp	1000	28.0 ± 0.0	93.3 ± 0.0^ns^	18.1
	500	25.0 ± 0.6	83.3 ± 1.9*	
	250	23.7 ± 0.3	78.9 ± 1.1*	
	125	19.0 ± 0.6	63.3 ± 1.9**	
	62.5	17.0 ± 1.2	56.7 ± 3.9***	
	31.25	16.0 ± 0.0	53.3 ± 0.0**	

Vincristine sulfate was used as positive control having IC_50_ value < 0.1 µg/ml; *<0.05, **<0.01, ***<0.001

*Rh.Cr* crude methanolic extract, *Rh.Hex* *n*-hexane fraction, *Rh.Chf* chloroform fraction, *Rh.EtAc* ethyl acetate fraction, *Rh.Aq* aqueous fraction, *Rh.Sp* saponins

^ns^ Non-significant

### Anti-angiogenic effect

**Fig. 4:**
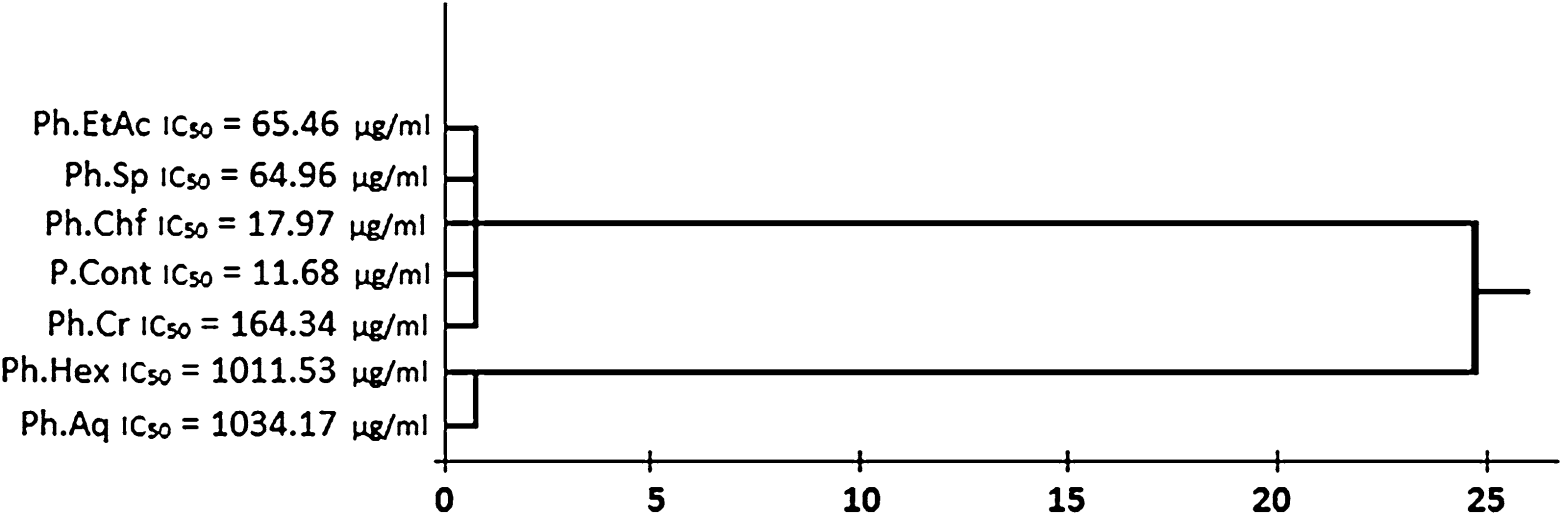
Cluster analysis and dendrogram based on IC_50_ values of various samples of *Rumex* *hastatus* in anti-angiogenic activity. *Rh.Cr* Crude methanolic extract, *Rh.Hex* *n*-hexane fraction, *Rh.Chf* chloroform fraction, *Rh.EtAc* ethyl acetate fraction, *Rh.Aq* aqueous fraction, *Rh.Sp* saponins, *P.Cont* positive control

**Fig. 5:**
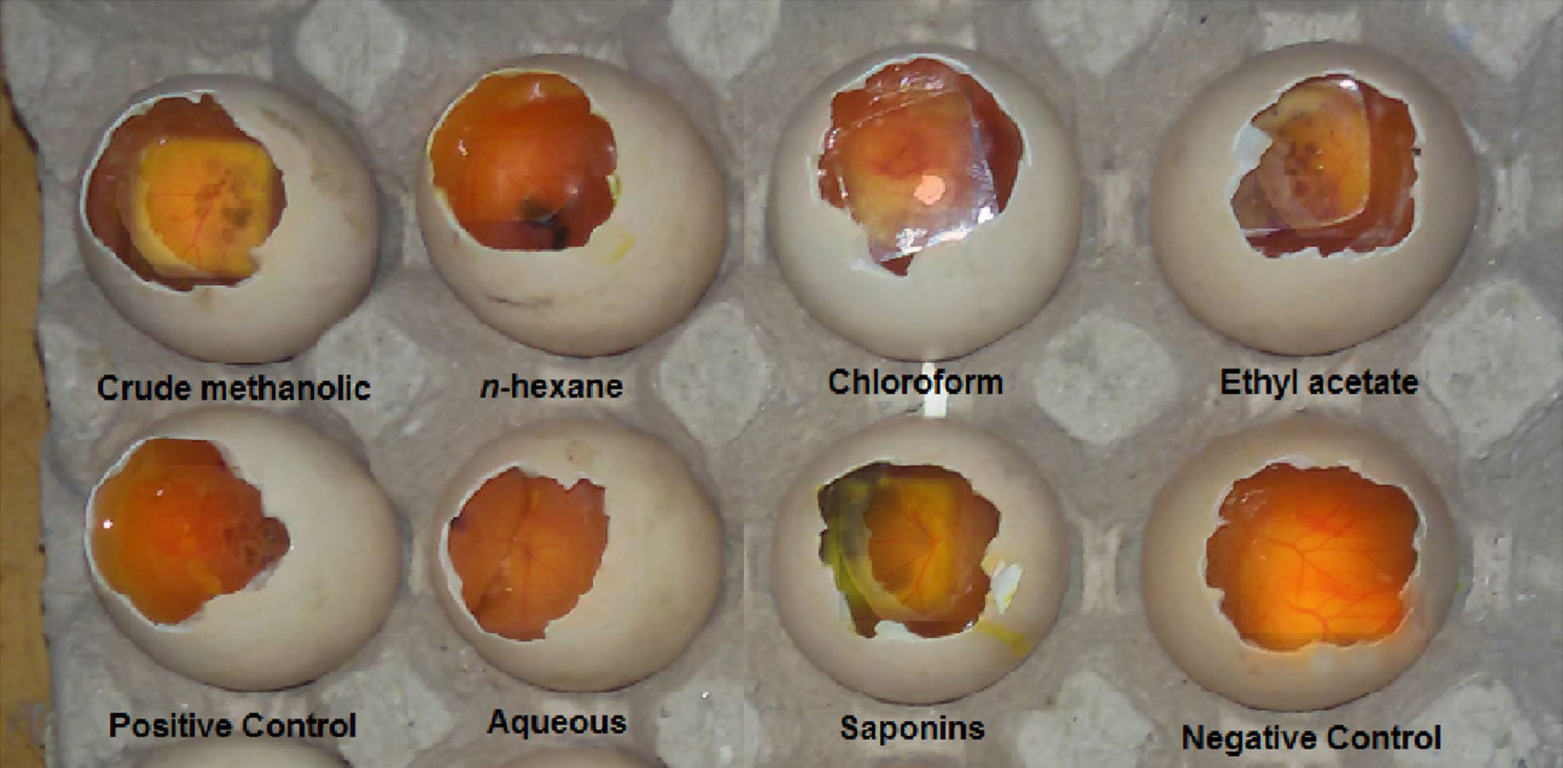
Anti-angiogenic activity of various samples of *Rumex* *hastatus* along with positive and negative control. Positive control CAM was applied with dexamethasone

Various samples of *R. hastatus* showed notable anti-angiogenic activity in CAM assay as presented in Table 2. The highest anti-angiogenic potential has been demonstrated by Rh.Chf in concentration dependent manner i.e., 56.7 ± 1.6, 59.3 ± 0.7, 64.7 ± 1.6, 68.3 ± 0.5, 74.3 ± 1.2, 81.6 ± 1.7 % at concentrations of 31.25, 62.5, 125, 250, 500 and 1000 µg/ml respectively with IC_50_ value of 17.9 µg/ml. Similarly, among the rest of the test samples, the Rh.Sp exhibited good inhibition of angiogenesis with IC_50_ value of 64.9 µg/ml. It is also obvious from the results that all the test samples showed dose dependent response. The result shown by positive control was almost comparable with the Rh.Chf. The order of activity recorded for various samples was Rh.Chf > Rh.Sp > Rh.EtAc > Rh.Cr > Rh.Hex > Rh.Aq with IC_50_ values of 17.9, 64.9, 65.5, 164.3, 1011.5, 1034.2 µg/ml respectively as given in Fig. 4 of cluster analysis. A summary of the experimental results in picture form is represented as Fig. 5.

**Table 2:** Results of anti-angiogenic assay of various samples of *Rumex hastatus*

Samples	Conc. 31. 25 µg/ml	Conc. 62.5 µg/ml	Conc. 125 µg/ml	Conc. 250 µg/ml	Conc. 500 µg/ml	Conc. 1000 µg/ml	IC_50_ µg/ml
Rh.Cr	39. 3 ± 0.7^***^	43.5 ± 0.6^***^	48.7 ± 0.8^***^	54.1 ± 0.2_***_	62.4 ± 0.3^***^	71.3 ± 1.3^***^	164.3
Rh.Hex	27.3 ± 1.3^***^	32.9 ± 0.5^***^	36.7 ± 0.8^***^	41.9 ± 0.3^***^	43.0 ± 0.2^***^	47.2 ± 1.1^***^	1011.5
Rh.Chf	56.7 ± 1.6^*^	59.3 ± 0.6^**^	64.7 ± 1.6	68.3 ± 0.5^*^	74.3 ± 1.2^*^	81.6 ± 1.7^*^	17.9
Rh.EtAc	43.3 ± 0.6^***^	49.6 ± 0.8^**^	55.3 ± 0.3^**^	61.0 ± 1.2^***^	67.3 ± 0.7^***^	72.3 ± 1.2^***^	65.5
Rh.Aq	11.6 ± 1.7	16.0 ± 1.1	23.3 ± 1.2	33.3 ± 0.3	37.0 ± 1.6	46.3 ± 0.4	1034.2
Rh.Sp	43.4 ± 0.9^***^	49.1 ± 1.4^**^	54.0 ± 0.6^**^	67.9 ± 0.5^**^	71.4 ± 0.3^***^	78.9 ± 1.0^**^	64.9

Dexamethasone was used as positive control with IC_50_ value of 11.68 µg/ml; data is represented as mean ± SEM (Standard Error Mean); * <0.05, ** <0.01, *** <0.001

*Rh.Cr* crude methanolic extract, *Rh.Hex* *n*-hexane fraction, *Rh.Chf* chloroform fraction, *Rh.EtAc* ethyl acetate fraction, *Rh.Aq* aqueous fraction, *Rh.Sp* saponins

### Antibacterial effect

Antibacterial activity carried out against *A.tumefaciens* demonstrated negligible activity of various plant samples. This activity was conducted to check that whether the test samples were active against specific strain or not. The antibacterial assay summarized in the Table 3 with minimum inhibitory concentrations (MIC) demonstrates that among the test samples, the Rh.Sp and Rh.Cr were active against the *A. tumefaciens*. The rest of the samples showed no antibacterial activity. Similarly, the result of Rh.Sp (MIC = 803.3 ± 8.8 µg/ml) and Rh.Cr (MIC = 1478.3 ± 4.4 µg/ml) were insignificant in comparison with the positive control (MIC for Ceftriaxone = 7.5 ± 1.4 µg/ml, MIC for Cefotaxime = 18.3 ± 0.6 µg/ml) which reveals that the *R. hastatus* is not active against *A. tumefaciens*. Briefly, the antibacterial assay of *R. hastatus* against *A. tumefaciens* does not reveal any significant antibacterial potential in comparison with positive control.

**Table 3:** Antibacterial activity of various samples of *Rumex* *hastatus* against *Agrobacterium* *tumefaciens*

Samples	Concentrations (mg/ml)	Zones of inhibition	MIC (µg/ml)
Rh.Cr	10	11.1 ± 0.4	1478.3 ± 4.4
	5	09.3 ± 0.7	
	2.5	NA	
	1.25	NA	
Rh.Hex	10	NA	–
	5	NA	
	2.5	NA	
	1.25	NA	
Rh.Chf	10	NA	–
	5	NA	
	2.5	NA	
	1.25	NA	
Rh.EtAc	10	NA	–
	5	NA	
	2.5	NA	
	1.25	NA	
Rh.Aq	10	NA	–
	5	NA	
	2.5	NA	
	1.25	NA	
Rh.Sp	10	15.5 ± 0.8	803.3 ± 8.8
	5	13.0 ± 0.4	
	2.5	11.9 ± 0.4	
	1.25	NA	
Ceftriaxone	10	21.5 ± 1.2	7.5 ± 1.4
	5	19.2 ± 0.3	
	2.5	17.5 ± 0.9	
	1.25	13.6 ± 0.7	
Cefotaxime	10	16.1 ± 0.4	18.3 ± 0.6
	5	11.3 ± 0.7	
	2.5	09.3 ± 0.7	
	1.25	NA	

Values are expressed as mean ± SEM

*Rh.Cr* crude methanolic extract, *Rh.Hex* *n*-hexane fraction, *Rh.Chf* chloroform fraction, *Rh.EtAc* ethyl acetate fraction, *Rh.Aq* aqueous fraction, *Rh.Sp* saponins

## Discussion

Anti-angiogenic and anti-tumor assays being non-invasive were carried out on fertilized eggs and potato discs respectively in our current investigational study. Herein, we have attempted to explore novel and natural source of anti-angiogenic and anti-tumor compounds due to high efficacy and low toxicity of natural products as compared to the synthetic compounds [5]. The purpose of employing the potato disc method and CAM assay was to get a rapid, economic and reliable results for anti-tumor and anti-angiogenesis potentials [30]. A dose dependent activity of various samples of *R. hastatus* was observed in both assays. The inhibitory effect of each sample has been expressed in percent and median inhibitory concentrations i.e., IC_50_. The assay is based on the hypothesis that anti-tumor drugs might inhibit the growth of tumors both in plant and animals, since some tumorigenic mechanisms are quite related in plants and animals [31]. A sound correlation has been demonstrated between the anti-tumor activity of bioactive compounds against 3PS in murine leukemia and potato tuber discs [32]. The inhibition of *A. tumefaciens* induced tumor is based on antimitotic activity and potato tumor assay can well demonstrate the 3PS activity as compared to 9 PS or the 9 KB cytotoxicity assay [33].

The angiogenesis process is described by migration of vascular endothelial cells from parental vessels, invasion through the matrix, proliferation and formation of capillary tube [34, 35]. While the anti-angiogenic agents bring about the inhibition of proteases, suppression of phosphorylation of receptors or disruption of endothelial tube formation [36, 37]. The anti-tumor potential exhibited by some samples of *R. hastatus* is stronger than several previously reported examples of various plants [10, 11]. The highest activity shown by methanolic extract of *Rumexdentatus* had been recorded as 56.6 %, which is lower than the least active sample of current investigation [38]. Likewise, the anti-angiogenic potential of *R. hastatus* is comparable with several plant species having strong anti-angiogenic potentials [13]. Moreover, the anti-angiogenic potential of various samples of *R. hastatus* is higher than the previously reported that daidzein and genistein [39].

In our current findings it has been demonstrated that various samples of *R. hastatus* were significantly effective to inhibit the tumor in potato disc model and angiogenesis in CAM assay. It has also been revealed that the test samples (except Rh.Sp and Rh.Cr) were inactive against the *A. tumefaciens* responsible for tumor induction in potato disc. It has also been observed that the anti-angiogenic and antitumor effects of certain fractions (Rh.Sp and Rh.Chf) were comparable with the positive controls as obvious from the Tables 1 and 2. Similarly, the cluster analysis of IC_50_ values can easily demonstrate the high potency of Rh.Sp and Rh.Chf in Figs. 2 and 4. Our study can be a part of serendipitous and intriguing findings for the upcoming researchers for the isolation of bioactive compounds from the highly active fractions of *R. hastatus*. The anti-tumor and anti-angiogenic activity of various samples of *R. hastatus* indicates the presence of bioactive principles within the highly active fractions. Based on the current investigational study, it is evidenced that the Rh.Sp and Rh.Chf might be good sources of bioactive principles which can impede the neo-vascularization and metastasis.

## Conclusions

Our results reveal that *R. hastatus* possesses strong anti-tumor and anti-angiogenic potentials. The chloroform fraction and crude saponins were most effective against tumor and angiogenesis. The Rh.Sp was negligibly active against *A. tumefaciens* but still can be considered for the antitumor research in the future. The most suitable target for the isolation of anti-neoplastic bioactive molecules will be Rh.Chf which was active in both activities and inactive against *A. tumefaciens*.

## Methods

### Chemicals and drugs

The solvents used in the current study were of pure HPLC grade (Sigma Aldrich). Vincristine sulphate, dexamethasone, cefotaxime and ceftriaxone were acquired from Sigma Aldrich distributors Peshawar Khyber Pakhtunkhwa Pakistan.

### Plant collection and extraction

The aerial parts of *Rumex hastatus* were collected in April from the proximity of University of Malakand. The plant was identified by Dr. Ali Hazrat, Plant Taxonomist, Department of Botany, Shaheed Benazir Bhutto University, Sheringal Dir Upper, KPK, Pakistan with voucher specimen 1015SA. It was shade dried for 15 days followed by grinding using a cutter mill. The powdered sample (7 kg) was subjected to maceration by dipping in 80 % methanol. After maceration for 15 days, it was filtered followed by evaporation of solvent under reduced pressure at 40 °C using rotary evaporator [40, 41]. Similarly, the crude methanolic extract (Rh.Cr) obtained was 400 g (5.7 %).

### Fractionation

The crude methanolic extract (Rh.Cr) having weight of 300 g was suspended in sufficient amount of water followed by fractionation with various solvents in separating funnel. The fractionation was started with less polar *n*-hexane (500 ml × 3), then with chloroform (500 ml × 3), then with ethyl acetate (500 ml × 3) and finally the aqueous fraction was obtained [42, 43].

Similarly, the fractions achieved were 19 (6.3 %), 21 (7 %), 29 (9.6 %) and 120 (40 %) g of *n*-hexane (Rh.Hex), chloroform (Rh.Cf), ethyl acetate (Rh.EtAc) and aqueous fraction (Rh.Aq) respectively.

### Extraction of crude saponins

For the extraction of crude saponins from *R. hastatus*, the plant powder having weight of 20 g was put in a conical flask and 100 ml of 20 % ethanol was added to the conical flask. The sample was heated at 55 °C in the water bath for 4 h with continuous stirring. After 4 h the sample obtained was filtered and the residue having greenish color was re-extracted with 200 ml of 20 % ethanol. The sample after extraction was heated until a concentrated volume of 40 ml was obtained. The sample obtained was transferred into a separating funnel and 20 ml of diethyl ether was added to it. After vigorous shaking, the separating funnel was put in a stand to get two layered sample. The lower layer was collected, which was aqueous layer while the upper diethyl ether layer was discarded. The aqueous layer obtained was diluted with 60 ml of *n*-butanol and the combined *n*-butanol extract was washed with 10 ml of 5 % sodium chloride solution. The final solution obtained was kept in a hot water bath until complete evaporation and the saponins obtained were dried in an oven yielding 1.3 g of crude saponin [44, 45].

### Anti-tumor assay

Suspension of *Agrobacteriumtumefaciens* 1 × 10^9^ colony forming units (CFU) was prepared in phosphate buffered saline. The suspension was standardized at 600 nm by an absorbance value of 0.96 ± 0.02 [46]. Stock solutions of plant samples were prepared and serially diluted to get the concentrations of 1000, 500, 250 and 125 µg/ml. The concentrations obtained were filtered into ependorf tubes using a sterile millipore filter (0.2 µ) to get sterilized. To each concentration 0.1 ml of *A.tumefaciens* broth culture were added and vertexed to mix well. Same procedure was followed for positive control i.e., vincristine sulfate while for negative control distilled water having 0.1 ml bacterial culture was used. Similarly red skinned potatoes were surface-sterilized for 30 min using 0.5 % sodium dichloroisocynnurate solution. These potatoes were then washed thoroughly using sterilized distilled water and aseptically dried. Discs having diameter of 1.5 cm and thickness of 0.5 cm were prepared from these potatoes using sterilized cork borer in aseptic environment. These discs were transferred into sterilized petri plates having solidified agar media. To each of the disc 50 µl of extract-bacterium mixture was applied using sterilized micropipette. Same procedure was followed for positive control while for negative control the distilled water having bacterial culture was applied on potato disc. The triplicate of each sample was used in this study. The petri plates were kept in the incubator (BOD incubator HYSC korea, model: Bl-81/150/250) at 25 °C for 18 days. After 18 days the surface of discs were stained with iodine solution and the number of tumors in each disc were counted with the help of binocular.

### Chick chorioallantoic membrane (CAM) assay

In the current study the fertilized chicken eggs were used to carry out CAM assay [47]. Fresh eggs were kept at 37 °C in incubator (BOD incubator HYSC korea, model: Bl-81/150/250) with narrow end down. The eggs were moved three to four times on daily basis. At fourth day, the eggs were examined and the head of embryo were encircled using a torch. One ml of albumen was sucked out from the narrow end of the eggs using 18-gauge hypodermic needle in order to move away the yolk sac and CAM from the shell. Similarly, the shell on the floor of the air sac was punched and peeled away. The thermanox coverslip already loaded with various concentrations of test samples was kept on the CAM surface so that the CAM surface come in contact with the test samples. After applying various samples the eggs were put back into the incubator. After 2 days a small volume of acetone and methanol (1:1) was infused into chorioallantois using 33-gauge needle. The CAM was carefully separated from the egg, the vessels were observed under microscope and number of vessels especially the vessels converging towards the center were calculated. For each test sample 18 eggs were used in this study. Dexamethasone was used as positive control while normal saline was used as negative control. The percent inhibition of angiogenesis was calculated using the following formula:

$$ {\text{\%  Inhibition}} = \frac{{{\text{CAM}}_{\text{ns}} - {\text{CAM}}_{\text{ts}} }}{{{\text{CAM}}_{\text{ns}} }} \times 100 $$

CAM_ns_ (number of blood vessels in CAM treat with normal saline); CAM_ts_ (number of blood vessels in CAM treated with test samples).

### Antibacterial assay

#### Preparation and standardization of inoculums

*Agrobacteriumtumefaciens* were cultured in petri plates having sterile nutrient agar media. Bacterial culture was transferred aseptically into sterile water for injection using wire loop. Suspension of bacterial culture was prepared having cell density of 1 × 10^6^ CFU/ml using McFarland standard. Suspension was standardized using UV–visible spectrophotometer and the standardization was maintained at 625 nm during the whole investigational study.

#### Well diffusion assay

Antibacterial activity of various samples *R. hastatus* were investigated against *Agrobacteriumtumefaciens* using well diffusion assay [29, 48]. Plates were prepared using nutrient agar media, labeled and test organism was inoculated under laminar flow hood aseptically. In each petri plate wells (5 mm) were bored using a sterile cork borer. Various concentrations (10, 5, 2.5, 1.25 mg/ml) of plant samples were prepared by serial dilution method. Test samples having volume of 100 µl were transferred into each well. After addition of test samples, the petri plates were incubated for 24 h at 37 °C. Cefotaxime were used as positive control. Similarly, the zone of inhibition of all the test samples was measured after incubation and the data obtained in triplicate was expressed as mean ±SEM.

#### Determination of minimum inhibitory concentrations (MICs)

For determination of MICs of various test samples, broth dilution method was followed. Stock solution was prepared for each sample having concentration of 5 mg/ml. From stock solution various dilutions were prepared using nutrient broth i.e., from 5 mg/ml to 0.61 µg/ml. To each sample 0.2 ml of bacterial suspension was added using a sterile micropipette. After inoculation, these samples were incubated at 37 °C for 24 h. After incubation, these samples were observed for turbidity and the minimum concentration, at which there was no bacterial growth was considered as the MIC of a specific test sample [49].

#### Statistical analysis

Two-way ANOVA followed by Bonferroni post test were applied for the comparison of positive control with the test groups. *P* values less than or equal to 0.05 were considered statistically significant. GraphPad Prism, SPSS software and Excel sheet were used to carry out Two-way ANOVA, cluster analysis and calculation IC_50_ values respectively. The standard error mean (SEM) were calculated at 95 % confidence intervals.
